# Humans or Dogs?

**Published:** 1920-03-27

**Authors:** 


THE PUBLIC WELL-BEING—(*continued*)

### Humans or Dogs?

The House of Commons is periodically treated to a speech from Sir Frederick Banbury in support of his own particular measure, the Dogs' Protection Bill, with the object of preventing vivisection on dogs. The usual desultory Friday afternoon discussion in the House gave place last week to a most interesting debate on a renewed effort by Sir Frederick Banbury to get his Bill pushed on, but it was talked out, and no vote was taken.

Strong opposition came from the medical members. The supporters of the Bill seemed mainly to rely on grounds of sentiment which deserve respect, but which hardly seem to weigh very strongly against the striking illustrations given by medical men of the inestimable value of vivisection. No man likes to think that the "friend of man" should be subjected to pain, but when it comes to a consideration of whether scientific investigation through vivisection of dogs can preserve thousands of human beings from terrible suffering and many forms of disease, it seems to resolve itself into the alternative of sparing the dog and indefinitely prolonging human suffering in the mass, or of using the dog, under proper safeguards, for the progress of medical science in many most vital respects.

It seems unreasonable, in any case, that the supporters

**THE PUBLIC WELL-BEING**—(*continued*).

of the Bill should merely have their eye on the protection of dogs; the return for 1913 showed that out of a total of 88,000 experiments on animals, only about 400 or 500 dogs were dealt with. It seems to show that the arguments are entirely sentimental. It would be difficult to imagine Sir Frederick Banbury working up annual indignation on vivisection of rats or any other disagreeable creature. But the chief reason why the dog is used in experiments, said Sir William Cheyne, is that more than any other animal his physiological processes approach closely those of man. When experiments were performed under an anæsthetic, and the dose of the anæsthetic was gradually increased until the animal died, there was no pain. Such experiments were in the majority. The idea that dogs suffered pain during experiments was due to memories of the early days, and was contributed to by the illustrations to be found in catalogues of instruments and appliances. Experiments which were followed by real pain must be very few. It was not necessary to abolish experiments on dogs in order to save them pain. The suggestion of cruelty was too foolish for words, and they could not afford to let slip the great opportunity of increasing their knowledge in the interests of the human race. With such an appalling death-rate from diseases of which nothing is known, we could not afford to give up any possible source of information, and Sir William expressed his belief that the solution of the problem of cancer would come through investigation on dogs.

Captain Loseby exposed the sentimental issue by pointing to the cruelty in various forms of sport, such as hunting, coursing, shooting of birds, and other things. Captain Elliott described the Bill as a pettifogging measure to obstruct the progress of medical science, and gave illustrations of the important results shortly to be expected in the treatment of heart disease by experiments on dogs, who were the only animals upon which experiments in relation to the heart could be carried out. One of the chief forms of heart disease was palpitation, and there were 20,000 sufferers among our pensioners alone. Great progress had been made towards finding a remedy, and in another year he believed success would be reached.

The debate throws a considerable light on the question, and if the average man with this definite medical expression of opinion before him reduces the question to individual considerations—will experiments on dogs promote the prospects of finding a cure for some frightful disease of a near relative or friend?—the answer in the great majority of cases, if not all, would surely be against the dog. Careful regulation and supervision are, of course, necessary, though it is difficult to understand why there should be any suspicion that the medical profession does not carry out this work with the same high motives which inspire it in all its work. Anyhow, vivisection is most stringently regulated, and the issue of licences is strictly confined to specially qualified and experienced investigators.

